# Low‐Field Actuating Magnetic Elastomer Membranes Characterized using Fibre‐Optic Interferometry

**DOI:** 10.1002/adfm.202301857

**Published:** 2023-09-17

**Authors:** Zhi Li, Joanna. M. Coote, Swathika Subburaman, Francesco Iacoviello, Kristopher Page, Erwin J. Alles, Polina Prokopovich, Ivan P. Parkin, Adrien E. Desjardins, Sacha Noimark

**Affiliations:** ^1^ Department of Medical Physics and Biomedical Engineering University College London London WC1E 6BT UK; ^2^ Wellcome / EPSRC Centre for Interventional and Surgical Sciences University College London London W1W 7TY UK; ^3^ School of Pharmacy and Pharmaceutical Sciences Cardiff University Cardiff CF10 3NB UK; ^4^ Electrochemical Innovation Lab Department of Chemical Engineering University College London London WC1E 7JE UK; ^5^ Department of Chemistry University College London London WC1H 0AJ UK

**Keywords:** fibre‐optic interferometry, magnetic actuation, magnetic elastomers

## Abstract

Smart robotic devices remotely powered by magnetic field have emerged as versatile tools for wide biomedical applications. Soft magnetic elastomer (ME) composite membranes with high flexibility and responsiveness are frequently incorporated to enable local actuation for wireless sensing or cargo delivery. However, the fabrication of thin ME membranes with good control in geometry and uniformity remains challenging, as well as the optimization of their actuating performances under low fields (milli‐Tesla). In this work, the development of ME membranes comprising of low‐cost magnetic powder and highly soft elastomer through a simple template‐assisted doctor blading approach, is reported. The fabricated ME membranes are controllable in size (up to centimetre‐scale), thickness (tens of microns) and high particle loading (up to 70 wt.%). Conflicting trade‐off effects of particle concentration upon magnetic responsiveness and mechanical stiffness are investigated and found to be balanced off as it exceeds 60 wt.%. A highly sensitive fibre‐optic interferometric sensing system and a customized fibre‐ferrule‐membrane probe are first proposed to enable dynamic actuation and real‐time displacement characterization. Free‐standing ME membranes are magnetically excited under low field down to 2 mT, and optically monitored with nanometer accuracy. The fast and consistent responses of ME membranes showcase their promising biomedical applications in nanoscale actuation and sensing.

## Graphical abstract

As shown above, the magnetic elastomer (ME) membrane is attached and fixed onto the cone‐shaped end of a glass ferrule. A cleaved single‐mode (SM) fiber is integrated and embedded within the ferrule channel pointing toward the ME membrane. The interferometric pattern formed due to the dual reflections upon the fibre tip and the ME membrane. The magnetic displacement of the ME membrane is measured by monitoring the change of the interferometric pattern in response to the length variations of the interferometric cavity.

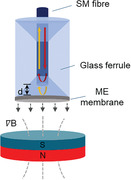

## 1 Introduction

Soft magnetic elastomer (ME) composites are a class of smart materials formed by embedding magnetic particles into non‐magnetic elastomer matrix host and curing into tailorable solid shapes. When exposed to the external magnetic field, the cured ME material can be accordingly magnetized and remotely actuated to rotate or translate in response to the magnetic torque or gradient force, or even deform due to the magnetic dipole–dipole interactions. Their superior mechanical stability, fabrication flexibility, and untethered actuation make them an ideal candidate for smart actuation or sensing, such as wearable electronics,^[^ ^1^ ^]^ soft robotics,^[^ ^2^, ^3^, ^4^, ^5^ ^]^ and microfluidic devices.^[^ ^6^, ^7^ ^]^ Nonetheless, the creation of ME composites with optimized magnetic responsiveness for the production of effective actuation devices is still challenging. First, the magnetic field at local sites is usually low due to the safety upper limit in the clinical context. It would decay rapidly and proportionally to the square of the distance away from the field source. Therefore, the magnetization and magnetic force are relatively small for sensing or actuation. Second, characterization and optimization of magnetic actuation devices under the as‐requested low field are rather challenging. This is mainly due to a lack of understanding about the composition dependent magneto‐mechanical properties as well as the availability of highly sensitive displacement analysis techniques. Development of highly responsive ME membranes, and in‐depth investigation of their magneto‐mechanical behaviors under low fields are critical and fundamental for smart device development.

Tremendous efforts have been made in the development of ME materials with high magnetic actuation performance. Soft magnetic particles with high susceptibility and magnetization as well as low coercivity and minimized remanence, e.g., carbonyl iron powder, have been intensively incorporated for the production of ME membranes. The rapid and non‐hysteresis responsive behaviors make them a better choice compared to their hard magnetic counterparts.^[^ ^8^ ^]^ To improve the mechanical flexibility and robustness of the ME composites, silicone elastomers, e.g., Ecoflex and Sylgard, or polymeric gels with high softness were employed to maximize its magneto‐mechanical responsiveness.^[^ ^9^ ^]^ Generally, high particle loading (up to 80 wt.% or 35 vol.%) is desirable to achieve strong magnetic responsiveness. However, the compromised mechanical flexibility should be considered as a result of the increased stiffness. The conflicting influences of particle loading upon magnetic and mechanical properties are yet to be discussed for overall actuating performance optimization.

To evaluate and optimize magnetic actuation capacity of ME membranes, highly sensitive displacement analysis techniques are crucial. Digital microscopes or cameras were commonly employed to carry out direct measurement of magnetic displacement of ME material.^[^ ^10^, ^11^, ^12^, ^13^ ^]^ However, they are limited in resolution when it comes to magnetic deflection or deformation within sub‐micrometre scale. Electrical techniques, including strain gauge are relatively more accurate and low‐cost.^[^ ^14^ ^]^ But it requires robust and stable contact with specimens, and raises concerns of extra deformation or unwanted electromagnetic interference. In contrast, optical displacement determination tools are based on acquisition and transduction of reflected signal, such as optical intensity or spectra,^[^ ^15^ ^]^ which are particularly advantageous in non‐contact and sensitive measurements of magnetic displacement. Using a white light speckle technique, Zhou and co‐authors were able to analyze the minor deformations of ME composite induced by magnetostrictive effects.^[^ ^16^ ^]^ Gong et al. developed a digital holographic interferometry system for the verification of local morphological transformations of ME composite under the external field.^[^ ^17^ ^]^ The aforementioned optical techniques enabled a closer look at tiny magnetic responses of ME composite and a better understanding of micro‐scale effects. Nevertheless, the high structural complexity of the sensing systems and the sample‐system integration issues make it difficult to be adapted and widely used for dynamic characterisation and optimization of ME material. Alternatively, commercial Laser Doppler Vibrometry (LDV) is a non‐contact and “point and shoot” technology best suited for direct measurements of displacement.^[^ ^18^ ^]^ It allows high frequency (up to GHz), high sensitivity (accuracy down to picometer), and fast speed 3D mapping in real‐time, which has been employed in a wide field, such as velocity monitoring and ultrasound mapping. However, it requires specialized and often costly equipment, and the miniaturization of such techniques^[^ ^19^ ^]^ and the line‐of‐sight^[^ ^20^ ^]^ limit in free‐space optics remain challenging for wider biomedical applications.

Efficient fabrication of free‐standing ME membranes with tailorable size is another problem to be addressed for the purpose of integration with sophisticated devices of various geometries. Developing ME membranes with good homogeneity and optimized performance from a highly concentrated and viscous composite presents a practical challenge. Here, we reported a simple and scalable doctor blading method to create thin and highly concentrated ME membranes. Through the employment of a tape template, the size and thickness of ME membranes were kept in good control. Ultra low‐cost natural magnetite powder and highly soft silicone elastomer with high affinity were employed to maximize the fabrication cost‐effectiveness as well as their magnetic actuation performance. Morphological characteristics and concentration dependent magneto‐mechanical properties were investigated. By introducing an optical fiber‐to‐membrane probe, ME membranes were secured and integrated with an established highly sensitive fiber‐optic interferometry system to enable dynamic magnetic displacement characterization with nanometer accuracy. Responsive and dynamic magneto‐mechanical behaviors of the ME membranes under low fields were accordingly studied and analyzed.

## 2 Results

### 2.1 ME Membranes and Membrane‐Ferrule Assembly

**Figure 1:**
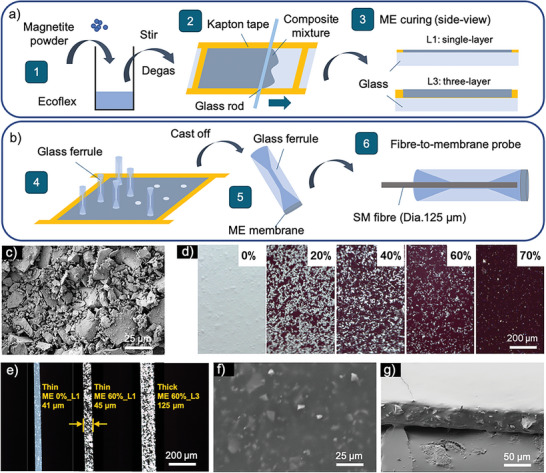
Schematic illustration of a) the two‐step doctor blading and ME composite curing within the Kapton template, b) the fabrication of the fibre‐membrane‐ferrule assembly. c) Scanning Electronic Microscope (SEM) image of the natural magnetic powder. Optical microscope images of the ME membranes of different particle concentrations and thicknesses (L1: single‐layer, L3: three‐layer) showing d) top‐view and e) cross‐sectional view. SEM images of ME 60%‐L1 membrane showing f) top‐view and g) side‐view.

ME membranes with a range of thicknesses and particle concentrations (See **Table** **1**) were successfully fabricated on glass slides using a two‐step doctor blading approach. During the process, the magnetite powder was mixed with Ecoflex elastomer, transferred, and bladed in custom‐made Kapton template, and eventually cured to form solid membranes under ambient conditions as depicted in **Figure** **1**a (for more details see Experimental Section). Mass ratios of the magnetic particles to the Ecoflex elastomer were kept at 0%, 20%, 40%, 60% and 70%, respectively. Photographic images of ME membranes of different concentrations were taken and shown in Figure S1a (Supporting Information). The thickness of ME membrane was controlled using a thickness‐varying Kapton template of single (L1) or three layers (L3) to match up the final thickness defined by the template “pool” depth. More images of free‐standing ME membranes of different sizes fabricated onto various frame structures can be seen in Figure S1d (Supporting Information).

**Table 1:** Fabrication parameters of ME membranes

Specimen	Particle concentration (wt.%)	Kapton template	Template thickness(µm)
ME 0%‐L1	0	1 layer (L1)	50
ME 20%‐L1	20	1 layer (L1)	50
ME 40%‐L1	40	1 layer (L1)	50
ME 60%‐L1	60	1 layer (L1)	50
ME 60%‐L3	60	3 layer (L3)	150
ME 70%‐L1	70	1 layer (L1)	50

An optical fiber‐to‐membrane probe design was proposed to achieve both efficient and stable integration with the fiber‐optic interferometry system. For that purpose, a ME membrane‐ferrule assembly was designed and fabricated as shown in Figure 1b. A double‐cone ended borosilicate glass ferrule (SHENGWEI, China) with a pore channel of around 127 µm was introduced to secure the connection with the inserted single‐mode (SM) fiber (dia.125 µm) and minimize its free tip vibrations (See Figure S1c, e–f, Supporting Information). Glass ferrules of two different cone end diameters (880 µm and 2000 µm) were customized and employed. The diameter of the attached ME membranes was therefore defined by the size of the ferrule cone end. This probe design enables both effortless lead‐in of cleaved fiber end and direct attachment of the free‐standing ME membrane.

### 2.2 Morphology of Magnetic Particles and ME Membranes

Morphological characteristics of the ME membranes were studied. Low‐cost and readily available natural magnetite powder was employed in this work. As seen in Figure 1c, it showed a dark black color, microscale size and irregular shape. After mechanical mixing with the Ecoflex elastomer, the magnetic particles were randomly dispersed within the polymer network and formed disordered agglomerates (See Figure 1d). A dense composite structure with less gaps was observed as the particle concentration increased. Upon cross‐sectional illumination (See Figure 1e), the ME membranes showed high internal uniformity across the whole volume. Cured Ecoflex without particle loading showed translucent color and smooth cross‐sectional morphology, in contrast to the darkened and mottled particle‐elastomer cross‐linking network of ME 60%. The employment of Kapton templates of different thicknesses enabled the successful fabrication of the ME membranes with two specific thicknesses. The final thicknesses of the ME membranes were double checked using digital micrometer and optical microscope as 43 ± 3 µm and 125 ± 5 µm, which were approximately close to the thicknesses of the Kapton templates: single‐layer and three‐layer tape (the thickness of single‐layer tape equals to 50 ± 4 µm). More morphological characteristics were revealed in top‐view SEM image (Figure 1f) where ME 60% showed a smooth surface with particles randomly oriented underneath. The surface roughness of all ME membranes was analyzed (See Figure S2, Supporting Information). Pure Ecoflex showed high smoothness and a low average roughness of around 17.2 nm, which is comparable to spin coated PDMS.^[^ ^21^, ^22^ ^]^ Surface profile scans of ME composite samples (Figure S2e, Supporting Information) showed that the inclusion of magnetic particles within the polymeric matrix increased surface roughness, resulting in a more “wavy” or “bumpy” membrane surface. Here, the average surface roughness of ME 20%, ME 40%, ME 60%, and ME 70%, were calculated to be 166.2 , 218.2 , 313.6 , and 610.1 nm, respectively. A significant increase of surface roughness and unevenness was statistically confirmed when particle loading reached up to 70%. More in‐depth internal images were acquired by cutting through the ME 60% membrane using a scalpel blade to expose its cross‐sectional structure (See Figure 1g). It was seen that magnetic particles with irregular shapes were fully immersed within the elastomer matrix. The solid and well‐connected network indicated good particle‐elastomer integration within such a heavily loaded composite.

### 2.3 3D Images of the ME Membranes

**Figure 2:**
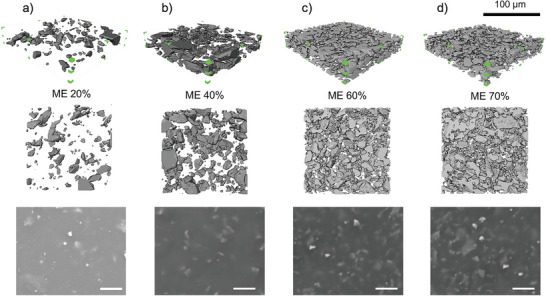
3D morphology and structure of the ME membranes with concentrations of a) 20%, b) 40%, c) 60%, d) 70%. The Micro‐CT images were processed in Avizo software to reconstruct their 3D structure. A representative sub‐volume (450× 450 × 85 voxel equivalent to 140 × 140 × 26µ m^3^, outlined in green color) was extracted from the entire volumetric tomogram, and displayed in tilted and front view, respectively. SEM images of ME membranes were accordingly displayed for parallel comparison of corresponding surface morphology (scale bar: 25 µm).

The volumetric microstructure of the ME membranes at micron‐scale was illustrated using a 3D X‐ray computed tomography (Micro‐CT) technique. To better visualize the internal structure of the ME membranes, Micro‐CT tests were accordingly performed. 3D rendering was created to distinguish the phases of both components. Therefore, the elastomer phase was deliberately made to be transparent (blank), in contrast with the dark grey particles owing to its much larger mass density. Poly‐dispersed size and irregular shape of magnetic particles observed in previous SEM images were once again confirmed in **Figure** **2**. The illustrated interior structure of all studied ME membranes showed the randomly orientated particles within the elastomer matrix as well as the largely improved particle number and connectivity in the ME membranes of higher concentration. SEM images of the ME membranes provided a comparative perspective of surface morphology, which were in good accordance with their 3D Micro‐CT images. There is a clear proportionality relationship between particle concentration and the particle‐elastomer ratio. Similarly dense structure was seen both in ME 60% and ME 70%.

ImageJ software (National Institutes of Health, USA) was used to quantify the volume fractions of magnetic particle and elastomer shown in **Table** **2**. ME 60% and ME 70% exhibited that the particle occupied volume percentage were 26 vol.% and 34.2 vol.%, respectively which were around the optimum concentration (30 vol.%) of ME material according to previous publications.^[^ ^23^, ^24^ ^]^

**Table 2:** The quantification of volume fractions of ME membranes

Specimen	Volume fraction (vol.%)	
	Magnetic particle	Elastomer
ME 20%	4.6	95.4
ME 40%	16.8	83.2
ME 60%	26.0	74.0
ME 70%	34.2	65.8

### 2.4 Magnetic and Mechanical Properties of the ME Membranes

**Figure 3:**
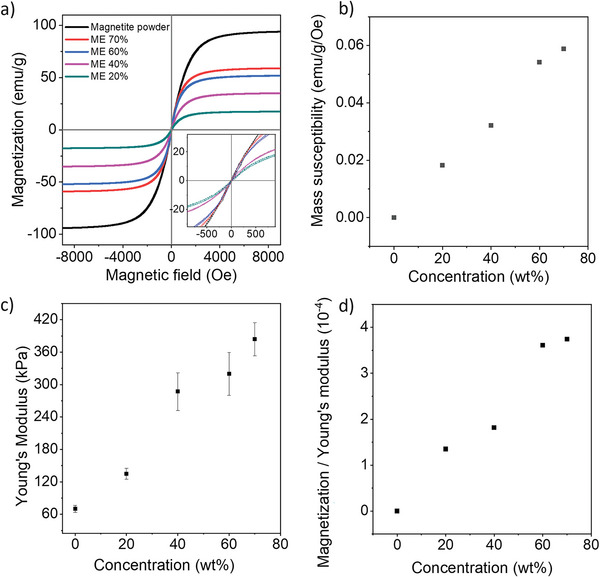
a) Magnetization curves of natural magnetite powder and the ME membranes. b) The correlation between magnetic particle concentration and mass susceptibility. c) Young's modulus of the ME membranes. d) The correlation between magnetic particle concentration and the ratio of mass susceptibility to Young's modulus.

By varying the loading ratios of the ME membranes, their magnetic and mechanical properties could be readily tuned. Vibrating sample magnetometer (VSM) was employed to characterize the magnetic properties of ME membranes with different concentrations. As seen in **Figure** **3**a, raw magnetite powder and ME membranes showed narrow hysteresis loops and typical ferromagnetic behaviors. The largest saturated magnetization was measured to be 92 emug^‐1^ for magnetite powder, and there is a proportional decrease for 20, 40, 60, and 70 wt.% ME membranes: 17.5, 34.9, 51.6, 58.9 emug^‐1^. By zooming in, low‐field magnetization behaviors of the ME membranes could be checked in detail. The magnetic remanences of all studied membranes were found to be less than 1 emug^‐1^ as well as the small coercivity, which suggested less hysteresis effects and better repeatability in magnetic responses. And there is a good linearity of magnetization against particle loading. Accordingly, the initial mass susceptibility χ_*m*_ upon particle loading was calculated and plotted in Figure 3b to show the linearly increasing magnetic responsiveness. Mechanical flexibility of the ME membranes was also quantified by measuring Young's modulus. As depicted in Figure 3c, Young's modulus increased with the increase of particle concentration. Higher stiffness and reduced mechanical flexibility in concentrated samples are in good accordance with previously reported work.^[^ ^25^, ^26^ ^]^ To evaluate the overall influences of particle loading upon both magnetic and mechanical properties, the ratio of initial mass susceptibility to Young's modulus of each ME membrane was introduced. As plotted in Figure 3d, the magnetic enhancement over‐weighed the mechanical reduction until concentration reached up to 60 wt.%, after which the ratio stayed fairly steady. It suggested that the improvement of magnetic responsiveness gained from the increased concentration was close to be balanced off by the corresponding stiffness enhancement. Taking further consideration of the fabrication challenge brought by highly viscous composite and its deteriorated quality including higher surface roughness and poorer particle‐elastomer integration, an upper limit of magnetic particle concentration around 60 wt.% should be considered.

### 2.5 Fiber‐Optic Interferometric Sensing System

**Figure 4:**
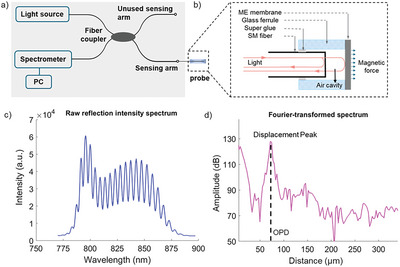
a) fiber‐optic interferometric sensing system (in grey block). b) Zoom‐in view of the ME membrane‐ferrule assembly for magnetic actuation. c–d) Reflective spectra of ME membrane c) before and d) after the inverse Fourier‐transform signal processing (OPD: optical path difference).

To characterize the magneto‐mechanical behaviors of the ME membranes, a self‐referenced fiber‐optic interferometric sensing system was developed based on previous studies of our group.^[^ ^27^ ^]^ A novel probe was added specifically in this work as shown in **Figure** **4**a,b. By inserting a cleaved fiber into the membrane‐ferrule assembly, an air cavity between the fiber end and the inner surface of the membrane formed. Dual reflected beams induced by the air cavity interfere and generate an interferometric pattern monitored by an optical spectrometer (See Figure 4c). Theoretically, this air‐cavity based interferometric pattern is closely dependent on the optical path difference (OPD) of two reflected beams:

(1) OPD=2nL,

in which n is the refractive index of air (n=1.0003), and L is the length of the air cavity. Any change of the cavity length (L+ΔL) would induce a change of the interferometric pattern:

(2) $$\delta =\Delta OPD\frac{2\pi }{\lambda }=4n\pi \left(L+\Delta L\right)/\lambda$$

where λ is center wavelength of the light source (λ= 830 nm). Inverse Fourier transformation (so‐called phase‐resolved low coherence interferometry) was used to translate the original reflected spectrum in the wavelength domain into the Fourier‐transformed spectrum in the distance domain. The Fourier‐transformed spectrum features an interferometric distance peak with its x‐axis value (OPD indicated by the black dash line) corresponding to the length of the air‐cavity (see Figure 4d). Consequently, by monitoring the complex argument (δ) of the inverse Fourier transformed spectrum (x‐axis position of the peak), the length variation of air‐cavity could be measured in real‐time so as to provide an instant indication of ME membrane deflection actuated by the external magnetic field.

### 2.6 Theory of Magnetic Actuation

**Figure 5:**
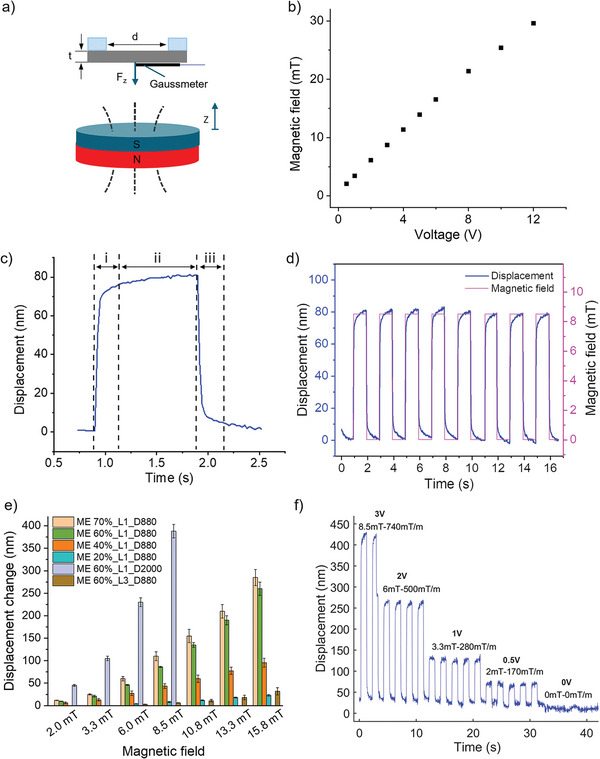
a) Schematic illustration of magnetic actuation mechanism of an edge supported circular ME membrane under external magnetic field. The gaussmeter was placed at the center point of the ME membrane for field monitoring during the measurements. b) Correlation between the modulated voltage against the generated magnetic field strength. c) One cycle of magnetic response of the ME membrane consisting of three stages: i) initial response, ii) quasi‐equilibrium, iii) recovery. d) Real‐time magnetic displacement of ME 60%‐L1‐D880 membrane under an 3V modulated pulse field (amplitude of 8.3 mT and gradient of 740 mTm^‐1^). e) Peak‐to‐peak magnetic displacement amplitudes of ME membrane with various particle concentrations, thickness, and diameter. D880 and D2000 represent two different diameters (880 µm and 2000 µm) of the ME membrane. f) Real‐time magnetic displacement of ME 60%‐L1‐D2000 under different voltage modulated magnetic fields (3, 2, 1, 0,5, and 0V).

A circular ME membrane bonded at the bottom of the ferrule was placed centrally over an electromagnet (around 5.5 mm) with the direction of magnetic field and gradient pointing upward (**Figure** **5**a). When exposed to the magnetic field, the ME membrane was easily magnetized along the same direction (z) and generated the magnetic pulling force uniformly across the whole body:

(3) $$M_{membrane}=\varphi M_{powder}$$

(4) $$F=\nabla B\cdot M_{membrane}$$

in which *M*_*membrane*_ and *M*_*powder*_ is the magnetization (emug^‐1^) of the magnetite powder, φ is the particle concentration of the ME membranes,∇*B* is the gradient of the applied magnetic flux density (tesla): *B* = µ_0_ · *H*, where µ_0_ is the vacuum permeability (4 × 10^7^ *T* · *m* · *A* ^−1^), and H is the magnetic field strength. Combining Equations (3) and (4), the pulling force can be further expressed as:^[^ ^4^, ^5^ ^]^

(5) $$F_{z}=V\cdot \nabla B\cdot M_{membrane}=V\cdot \mu_{0}\cdot \left(\frac{\Delta H_{z}}{\Delta z}\right)\cdot M_{membrane}$$

where V is the effective volume of the ME membrane, *M*_*membrane*_ is the magnetization of the ME membrane per volume (*emu*/*cm* ^3^), $\frac{\Delta H_{z}}{\Delta z}$ is the applied field gradient between the upper and lower surface of the membrane, and Δz corresponds to the thickness (t) of the membrane.

In response to the uniformly distributed magnetic pulling force, the circular and edge‐supported ME membrane was forced to deflect and produce resisting elastic force to prevent being stretched till an equilibrium was reached. Due to the symmetrical alignment of the ME membrane and the field, this deflection would be restricted to be out‐of‐plane and maximized at the center of the membrane. The maximized displacement can be calculated as:^[^ ^28^ ^]^

(6) $$\omega_{0}=\frac{p_{z}r^{4}}{64G}$$

(7) $$p_{z}=\frac{F_{z}}{S}$$

in which *p*_*z*_ is magnetic force pressure (*Nm* ^−^^2^), r is the effective radius of the deflecting membrane (r= d/2, d is the diameter of the membrane), S is the effective area of the membrane, G is the flexural rigidity:

(8) $$G=\frac{Et^{3}}{12(1-v_{0}^{2})}$$

where E is Young's modulus of the ME membrane (*Nm* ^−^^2^), *v*_0_ is Poisson's ratio assumed to be a constant value 0.49 for the ME membranes. Combining Equations (6)– (8), the maximized displacement at the centre point of ME membrane can be finally written as:

(9) $$\omega_{0}=\mu_{0}\frac{\Delta H_{z}}{\Delta z}M_{membrane}\left[\frac{3r^{4}\left(1-v_{0}^{2}\right)}{16Et^{2}}\right]$$

Aforementioned results provided a good guidance in optimizing magnetic actuation capacity by taking into account the correlation between the membrane parameters and the predicted displacements. According to Equation (9), ME membranes with smaller thickness, larger diameter, lower Young's modulus, and higher magnetization are superior to achieve larger magnetic actuating capacity.

Before magnetic actuation tests, theoretical calculation and ANSYS simulation (version 19.0, see **Table** **3** and Figure S3, Supporting Information) were performed to mimic the magnetic deflection of a circular free‐standing ME membrane (ME 60%‐L1‐D880), which was attached to an open end of the ferrule. A circular and edge‐supported disk model was therefore built with external force loading uniformly applied upon its surface to induce disk deflection. Magnetic pulling force was employed as the actuation source in this case, which was theoretically calculated by following Equation (5). According to ANSYS simulation, the magnetic deflection of ME 60% under a 3V field is as large as 106 nm. The corresponding value obtained from the theoretical calculation was around 200 nm. The big discrepancy between both results may be due to the unresolved boundary condition: edge clamped or edge simply supported in ANSYS, which however would make a huge difference in theoretical calculation.^[^ ^28^ ^]^ To verify, the magnetic deflection of an edge‐clamped membrane was accordingly calculated and obtained to be 54 µm (See Table S1, Supporting Information). It is indicated that the unresolved boundary condition with two extreme limits may lead to a range for the final displacement. Therefore, in ANSYS simulation or under experimental conditions, the displacement may vary in a range between 54 and 200 µm. (More information refers to the discussion in Table S1, Supporting Information) Nevertheless, by conducting the theoretical calculation and ANSYS simulation, the influences of material parameters upon overall actuation capacity were better understood, which provides good guidance in the estimation and optimization of the final displacement of the ME membranes.

**Table 3:** Theoretical modeling of the ME membrane deflection using ANSYS

Parameters	Description	Quantity	Unit
φ	Particle concentration	60	%
*d*	Diameter	880	µm
*E*	Young's modulus	320	kPa
*t*	Thickness	45	µm
*B*	Magnetic field	8.3	mT
$\frac{\Delta H_{z}}{\Delta z}$	Gradient along z direction	740	mT/m
*F*	Magnetic pulling force	0.181	µN
ω_1_	Theoretical deflection (edge simply supported)	200	nm
ω_2_	ANSYS simulated deflection	106	nm

### 2.7 Magnetic Actuation Characterization

Magnetic displacements of the ME membranes were characterized using the fiber‐optic interferometric sensing system. In this work, square‐wave magnetic field produced by an on‐off voltage‐modulated electromagnet was employed. Under the pulse field modulation, the stabilized displacement level could be constantly maintained during each cycle period, and readily calculated for performance comparison and optimization. The field strength was linearly tuned by the applied voltage of the power supply via the LabVIEW programme as plotted in Figure 5b. A single cycle response of the ME 60% membrane (L1‐D880: thickness of 43 µm and diameter of 880 µm) was recorded in Figure 5c. It showed a large peak‐to‐peak displacement of around 86 nm under 3V (field amplitude and gradient peaked at 8.3 mT and 740 mT), which is very close to the result of ANSYS modeling (106 nm). Variations of the displacement may rise as the membrane could be slightly off‐centre or heavily sealed on the ferrule end, and led to a reduced diameter. There is a high signal‐to‐noise ratio of the response curve as well as a low background noise of the sensing system around 2.3 nm (See Figure S4, Supporting Information). Generally, magnetic response of the ME membranes comprises three stages: i) initial response, ii) quasi‐equilibrium, iii) recovery. The first stage featured a rapid displacement as the field was turned on following with an outward deflection of the ME membrane. Afterwards, a quasi‐equilibrium stage appeared as the displacement slowly saturated due to the balance of the magnetic pulling force and the elastic resistant force. A slow ramping curve due to the creeping effects was observed before reaching up to the final steady stage. It probably results from the magnetic hysteresis or viscoelastic properties of the ME membranes. Once the magnetic field was turned off, a rapid recovery happened as the membrane was drawn back to its initial position. The magnetic response speed of the ME membranes was analyzed by fitting the response curve (initial response stage) according to an exponential function y = a + b*exp(t/τ), where τ is the time constant corresponding to the magnetic response time.^[^ ^27^ ^]^ The response time of ME 60% was fitted to be as low as 0.0236 s (See Figure S5, Supporting Information), which indicated a rapid magneto‐mechanical response. Dynamic magnetic actuation and characterisation of ME membranes under high frequency were performed by reducing the cycle period of the pulse field (See Figure S6, Supporting Information). The magnetic displacement can be consistently achieved and maintained in a fast manner as the period was tuned down to 0.2 s (5 Hz). Higher excitation frequency (pulse period: 0.08 s, frequency: 12.5 Hz) caused slight displacement reduction as a fully stabilized displacement (the second quasi‐equilibrium stage) could not be reached before recovering. Further increase of excitation frequency caused discontinuous and distorted responses as ME membrane failed to follow the field modulation until completely out of phase, although the displacement oscillations were still optically witnessed. Therefore, high frequency excitation of the ME membranes based on faster field generator and spectrometer is to be more in‐depth explored in future work. To demonstrate the good repeatability of the magnetic actuation, cycles of magnetic responses were recorded in real time as shown in Figure 5d.

ME membranes with various particle loading, coating thickness and diameter were tested under low magnetic field. As seen from Figure 5e, the ME membranes showed proportionally increased displacements against magnetic field and particle loading. ME 60% and ME 70% exhibited larger and comparable responses compared to the less loaded ME membranes. Increasing thickness of ME 60% from 43 µm (L1) to 125 µm (L3) caused a sharp reduction of displacement from 86 to 6 nm under 3V (amplitude of 8.5 mT and gradient of 740 mTm^‐1^). Displacements of thick ME 60% (125 µm) under a field less than 8.5 mT were too small (around 3 nm) and beyond the detection limit of the sensing system. On the other side, a huge improvement in displacement was observed when the diameter grew from 880 to 2000 µm. The magnetic displacements of ME 60%‐L1‐D2000 were found to be 45, 105, 230, and 388 nm under 0.5, 1, 2, and 3V, which exhibited a non‐linear relationship between the displacement and the applied voltage. This may be due to the quadratic rise of the magnetic loading force induced by the combined effects of the field dependent magnetization and the magnetic gradient (according to Equation (4)). It is worth mentioning that the magnetic displacements of ME 60% under higher field (> 10.8 mT) were not shown for comparisons due to the phase wrapping effects. This happens when the transient displacement change (between two sampling points) upon a sharp field pulse exceeds a limit when the interferometric phase change (Δδ) is over π. Owing to the periodicity of optical interference, displacement induced phase change over π could not be optically recognized. Therefore, more sampling points are needed to avoid any sharp changes of displacement so that continuous recording could be achieved. A sensing system with faster data acquisition and processing would be helpful. Alternatively, steady varying field, e.g., step‐wise varying field, can be applied with minor field change between steps from one level to another and enables consistent displacement measurements under large field variations (See Figure S7, Supporting Information). Figure 5f showed the real‐time magnetic responses of ME 60%‐L1‐D2000 under different fields (less than 10 mT). Excellent repeatability and responsiveness were demonstrated under field as low as 2 mT despite slight zero‐drifting effects caused by the vibration or expansion of the membrane.

## 3 Discussion

This work presented a straightforward and scalable two‐step doctor blading fabrication approach enabling the creation of highly concentrated ME membranes using low‐cost and readily available natural magnetite powder. It allows a proper thickness control of thin ME membranes (tens of µm) defined by the tape template while maintaining the good homogeneity when fabricated from a highly viscous composite. In contrast, other traditional methods are all restricted in this scenario. For instance, dip‐coating and spray coating are suited for diluted and less viscous mixture; moulding^[^ ^24^, ^29^ ^]^ is more efficient in fabricating samples with scalable thickness up to millimetre; spin‐coating^[^ ^30^, ^31^, ^32^ ^]^ are challenging in production of size‐scalable and highly concentrated membrane. The fabricated ME membranes show dense and well‐integrated particle‐polymer structures in SEM and Micro‐CT images even when particle concentration increases over 60 wt.%. There is a good wettability of magnetic particles surrounded by the elastomer. Contact angle tests were accordingly performed to check their surface hydrophilicity (See Figure S8, Supporting Information). As a result, both raw magnetite powder and the ME membranes showed similarly large contact angles (around 107 degrees), which explained the high hydrophobicity and good affinity between the magnetic particle and the elastomer. The ME membranes demonstrated uniform thicknesses in all studied concentrations. This is favorable for reflective light acquisition and high‐quality optical interference. Surface roughness analysis of ME membranes with different particle concentrations was undertaken and showed that an increased particle loading corresponded to an approximately linear increase in surface roughness up until ME 70%, at which point the elastomer surface integrity deteriorated with a significant increase in surface roughness and unevenness. (See Figure S2, Supporting Information) Nonetheless, the surface roughness of all studied ME membranes was still in sub‐micrometre scale. Benefiting from the low‐coherence interferometry scheme and its low requirements of optical reflection, the developed fiber‐optic sensing system demonstrated a good signal‐to‐noise ratio as well as a low limit of displacement detection (around 3 nm). ME 70% showed observable surface inhomogeneities with bumpy traces under profilometry examination, contributing to an extremely rough surface, and highlighting the reduced integrity between the magnetic particle and the elastomer. It is worth mentioning that the fabrication of ME membranes with concentrations over 70 wt.% is extremely challenging. Largely increased viscosity and poor conditions of composite caused by reduced coverage of elastomer upon magnetic particle make the mixing and blading process more difficult. Using current approach, 70 wt.% appears to be a practical limit for the production of ME membranes with good quality and high reproducibility. To improve the particle loading even further, surface modification of magnetic particles or physiochemical affinity of elastomer are crucial.

The ME membranes exhibit combined merits of both magnetic and elastomeric components, and feature tuneable magneto‐mechanical properties. The low‐cost natural magnetite powder shows typical soft ferromagnetism, such as large susceptibility and minimized hysteresis loop. It is especially beneficial for fast and reversible magnetic actuation compared to their hard magnetic counterparts. By increasing magnetic particle concentration, the magnetization of ME membrane was proportionally increased to maximize their magnetic actuation capacity at the highest concentration 70 wt.%. On the other side, high mechanical flexibility of ME membranes was achieved through the employment of super soft Ecoflex^*TM*^ 00–30 elastomer (low 100% modulus of around 70 kPa). Although it gets stiffer as particle loading increases, Young's modulus of ME 70% is still maintained as low as 0.320 MPa, which is much lower than commonly used silicon elastomers, i.e., Sylgard 184 (a few MPa). The overall effects of particle loading on both properties have been investigated and the trade‐off effects of concentration are close to be balanced off when it is over 60 wt.%. Taking into account both fabrication applicability and combined performance, 60–70 wt.% would be the optimal concentration range using the current approach.

Efficient actuation of ME membranes under relatively low magnetic fields is critical and challenging for developing smart devices with low energy consumption. ME membranes are usually weakly magnetized under these circumstances and produce barely detectable magnetic responses. It puts forward high demands on the development of highly accurate displacement characterization tools for performance optimization. A highly sensitive, robust and low‐cost fiber‐optic interferometric sensing scheme was first applied to solve this problem. It comprises inexpensive optical components such as a SLED light source, a fibre‐optic coupler, and a spectrometer. It allows rapid detection of field induced displacement by optically tracking the variations of the cavity based interferometric pattern through the phase resolved low coherence interferometry. All optical, high resolution and non‐contact sensing scheme make it an ideal tool for magnetic actuation characterization. A key challenge of this technology is to achieve a high‐quality alignment of SM fiber to ME membrane, and stabilize the air‐cavity structure for nanometer‐scale displacement measurement. Subtle misalignment resulting from unstable end connection or minor vibration of the fiber tip, can cause largely angled optical reflection and increased background noises. It eventually leads to much reduced sensitivity. According to ANSYS simulation, edge‐supported circular membrane under uniformly loaded pulling pressure would generate symmetrical displacement, which decays rapidly from the maximum at the center point to zero at the edge. Consequently, large errors can be produced due to tiny fiber tip movement. To address this issue, a double‐cone glass ferrule was employed to easily guide and secure the connection in position. The matching size of the ferrule pore channel and the SM fiber only allows axial translation of the fiber within the ferrule but leaves no room for transversal movement. Once the ME membrane was attached to the cone‐shaped open end of ferrule using silicon adhesive, the SM fiber would auto‐centre itself and align perpendicularly toward the planar surface of the free‐standing ME membrane. This integrated probe design is simple in structure, easy to operate and efficient for stability improvement. Based on the current probe design, the ME membranes were magnetically actuated and optically tracked under low fields that opens up avenues to magnetic response characterization in microscale.

Using the aforementioned technique, the fabricated ME membranes were magnetically actuated and optically characterized. Unlike ME composite made from hard magnetic fillers, soft ME composite is often less efficient in actuation due to its much weaker magnetization. However, it is often preferred in practical applications because it becomes nonmagnetic and safe when the external field is removed. By increasing the loading ratio up to a limit (60–70 wt.%), the magneto‐mechanical performance can be saturated and maximized based on the aforementioned discussion. Further improvement in magnetic actuation performance may be achieved by tailoring ME membranes into bigger and thinner pieces. According to Equation (9), the magnetic displacement of the ME membrane scales up with 4th power of diameter and shrinks down with the square of the thickness. As an example, a real‐time video was recorded (See Supplementary Video) when a large piece of ME 60% (45 µ m in thickness) was actuated under a strong pulse field (20V, 48 mT). The ME membrane was fabricated and attached to the open of a round glass petri dish (dia. 35 mm), and actuated to displace back and forth in millimeter scale. Alternatively, high performance ME membranes can be fabricated to be ultra‐thin (micron‐meter) using corresponding thinner tapes. In this case, the mechanical strength of the ME membrane should be carefully considered.

Being capable of measuring displacements in nanometre scale, the newly developed fiber‐optic sensing system is of broad interest in solving existing scientific problems. For instance, it would be useful in characterizing microscale magnetic deformation of ME composite so as to more clearly clarify the mechanism of magnetorheological effects. Alternatively, it can be employed to calibrate the magnetic pumping (membrane based) capacity of the ME membrane based microcapsules under low fields for quantitative drug delivery and release at remote local sites. Last but not least, owing to its miniature size, all‐optical structure, and high sensitivity, the fiber‐optic magnetic sensing system would be interesting to be developed further as a smart device combining both actuation and sensing, which is promising for medical device localization and tracking in MRI context.

## 4 Conclusion

In summary, we have demonstrated a highly cost‐effective and well‐controllable approach to fabricate low‐cost, centimetre‐scale, thin, and heavily loaded (up to 70 wt.%) ME membranes for magnetic actuation under low field. A highly sensitive fiber‐optic sensing method was first applied to characterize their magnetic actuation performance. The ME membranes showed tunnble magnetic and mechanical properties. Magnetic and mechanical characterizations have been conducted to verify the balanced conflicting influences of concentration upon magnetic actuation performance and the optimized particle loading range (60–70 wt.%). Upon magnetic modulation and optical characterization, the ME membranes exhibited large, fast, and reproducible magnetic response. In future, more fundamental and comprehensive studies of the magnetic responsive behaviors of ME membranes could be conducted, which will be beneficial for developing miniaturized magnetic actuators or sensor for wide biomedical applications, such as smart robotics or MRI‐guided interventions.

## 5 Experimental Section

### Preparation of the ME Membranes

ME membranes were fabricated on glass‐slide substrates through a two‐step approach, which mainly includes two processes: ME composite preparation and doctor blading. First, soft ferromagnetic particles (natural magnetite powder, Inoxia, UK) and silicone elastomer (Ecoflex^*TM*^ 00‐30, Smooth‐on, USA) were manually mixed in certain weight ratios followed with 3 min ultrasonication to release air bubbles generated inside the composites. A specific fabrication template was designed for doctor blading of ME membranes. Typically, a centred “pool” structure was fabricated on the glass‐slide substrate by attaching Kapton tape on the top and its four sides to leave the center area surrounded by the edge tape. Doctor blading was performed by smoothly translating a glass rod (dia. 2 mm) through the “pool” template, which was previously covered with ME composites, and finally created a uniform and homogeneous ME membrane in the “pool”. The ME membranes were maintained under ambient conditions for around 4 h to allow the particle‐silicone composite to fully cure.

### Fabrication of ME Membrane‐Ferrule Assembly

The customized glass ferrules were vertically placed onto the cured ME membrane on the glass slide substrate using thin silicone adhesive (MED1000, Nusil, USA). After 2 h curing, the ME membrane‐ferrule assembly was removed and cast off using a scalpel blade from the glass slide, which created a one‐end sealed ferrule assembly that could be easily integrated and connected with the optical fiber system.

### Morphology Characterisation

The SEM images of the raw magnetite powder were taken prior to the preparation of ME membranes to double check the shape and size of the magnetic particles. Top‐view microscope images were used to study the surface morphology and particle homogeneity of ME membranes of different concentrations while cross‐sectional images were used to provide an indication of membrane thickness and particle concentration. Especially, side‐view SEM images were taken to inspect in detail the surface state of ME membranes with high magnetic particle concentration (60 wt.%) as well as its particle‐elastomer integration. The optical images were acquired using a stereomicroscope (M125, Leica, UK), and SEM tests were performed using a field‐emission scanning electron microscope (SEM, XB1540, Carl Zeiss, Germany). For each SEM test, an additional thin gold layer was coated on the top of the samples beforehand using a thermal evaporator (Auto 306, Edwards, UK) to improve the surface conductivity for SEM image studies.

### 3D Structure Characterization

The internal structure of the ME membrane was checked using a lab‐based X‐ray micro‐scale CT scanner (Micro‐CT, ZEISS Xradia 620 Versa, Carl Zeiss Inc., Pleasanton, USA), which employed a polychromatic tungsten target source with the voltage set at 120 kV, a 40X lens and a 2048 × 2048 CCD camera detector (binned 2) to achieve a voxel resolution at 312 nm with a field of view (ca. 312 × 312µ m^2^). This was a non‐destructive microscopy technique that allows see‐through visualization of 3D structure and quantitative analysis of composite materials. ME membrane samples for tests were prepared in a disk shape with a diameter of 20 mm and a thickness of 50 µm. The Micro‐CT image data was processed in Avizo (2019.4, Thermo Fisher Scientific, USA). A sub‐volume of 450 × 450 × 85 voxels (0.3125µ m/voxel pixel) in length, width, and height (ca. 5.25 · 10^5^µm^3^ in volume) was extracted after correction of sample tilt for further image reconstruction, segmentation, and quantification. An unsharp mask filter was applied to sharpen the edges of the particles without increasing the noise. A two‐phase binary segmentation method was employed based on the gray scale values of both components (particles and elastomer) using a combination of threshold method for 3D rendering visualization. Despite the inevitable estimation inherent to the use of filters for network extraction, this method provided a direct approach to study the spatial distribution and connectivity of the magnetic particles dispersed within the elastomer matrix as well as their relative ratios.

### Mechanical Characterization

Young's modulus measurements of the ME membranes were carried out through nanoindentation according to a published method by Polina Prokopovich et.al.^[^ ^33^ ^]^ Briefly, an atomic force microscope (AFM, XE‐100, Park Systems, South Korea) was employed for adhesion force measurements using a cantilever (Veeco, ORC8‐10, tip radius of 15 nm, nominal spring constant *K*_*cantilever*_ of 0.05 Nm^‐1^). Therefore, Young's modulus of the samples was calculated by following the formula:

(10) $$F=\frac{2E}{\pi }\frac{\tan\alpha }{\left(1-\nu_{0}^{2}\right)}\delta^{2},$$

where F is the adhesion force recorded by AFM, E is Young's modulus, α is the semitop angle of the AFM tip (constant value 18°), ν_0_ is the Poisson ratio (assumed to be 0.49 for all ME membranes), δ is the indentation depth (a fixed value for each measurement). Twenty measurements were performed on different locations of each membrane to allow assessment of surface homogeneity (spatial variation) and avoid measurement errors by scaling up the sampling area range.

### Magnetic Characterization

Magnetization curves of the magnetite powder and the ME membranes were carried out at room temperature using a physical property measurement system (PPMS 14T, Quantum Design, USA) with a maximum applied field of µ_0_H= 1 T. The measurements were performed by applying the field parallel to the surface plane of the membrane samples.

### Fiber‐Optic Interferometric Sensing System

A fiber‐optic interferometric sensing system was developed and established as a self‐referenced Michelson interferometer, which was previously used for physiological temperature and pressure sensing^[^ ^27^ ^]^ in the group. As shown in Figure 4a, the broadband light with a central wavelength of 830 nm, spectral width of 62 nm and output power of 1.25 mW was emitted from a superluminescent light source (SLED, SLD‐MS‐351‐MP‐SM, Superlum, Ireland), passed through an in‐line attenuator (VOA‐850‐APC, Thorlabs, USA) and a 2×2 fiber‐optic coupler (50:50, TW850R5A2, Thorlabs, USA) and reflected from a sensing arm (the other arm was currently unused and capable of multiplexed sensing) to a compact broadband spectrometer (Flame‐S, Ocean Optics, USA) with an acquisition time of 1 ms. A SM fiber (780HP, Thorlabs, USA) was then spliced and connected to the sensing arm of the fiber‐optic interferometric sensing system and left the other fiber distal end free for integration with the ME membrane‐ferrule assembly. Being stripped of buffer coating, cleaned with isopropanol (IPA) and cleaved perpendicular to its optical axis, the SM fiber end was steadily aligned and smoothly translated into the membrane‐ferrule assembly using a high‐precision 3D translation stage (PT3A, Thorlabs, USA) till an air cavity formed between the fiber tip and the internal membrane surface.

The raw reflected spectrum of the cavity was first acquired by the spectrometer and processed in a customized program written with LabVIEW (National Instruments, USA). The overall sampling rate was up to 250 Hz. The complex argument of the inverse Fourier‐transformed spectrum at the x‐axis value of the distance peak was tracked and calibrated into small variations in “displacement” of the air cavity, and displayed in real‐time. To achieve a high signal‐to‐noise ratio, the initial air‐cavity length was readily tuned (0–1500 µm) by fine control of the length of the SM fiber embedded inside the membrane‐ferrule assembly until a strong and sharp peak appeared, which symbolized an optimized light‐circuit alignment and strong interference. Using this method, the membrane displacement could be precisely monitored in real‐time by monitoring the distance peak shifting in the inverse Fourier‐transformed spectrum.

### Magnetic Actuation

Magnetic actuation tests were performed under the magnetic field produced by an electromagnet (P50/27, Sourcingmap, China) powered using a programmable power supply (E36103A, Keysight, UK). Either static or dynamic magnetic field with a tuneable time period (> 0.02 s), frequency (< 50 Hz), and amplitude (0– 50 mT) could be generated by modulating the voltage output of the programmable current generator. For simplicity, an on‐off pulse field was employed for magnetic actuation by modulating periodic input voltages of the electromagnet. The field was generated along the out‐of‐plane symmetrical axis of the electromagnet, which had been mapped out in Figure S9 (Supporting Information) using a commercially available gaussmeter (GM08, Hirst Magnetic Instruments, UK). During the measurement, the ME membrane was located perpendicular to the field direction of the magnet and aligned with their center axes coinciding symmetrically along the z‐axis as shown in Figure 5a. The displacement peak (inverse Fourier‐transformed spectrum) was recorded in real‐time accordingly while the magnetic field was applied.

## Conflict of Interest

The authors declare no conflict of interest.

## Supporting information

Supporting Information

Supplemental Video1

## Data Availability

The data that support the findings of this study are available from the corresponding author upon reasonable request.
