# Supplementary material for: Low‐Field Actuating Magnetic Elastomer Membranes Characterized using Fibre‐Optic Interferometry
Source: Adv Funct Mater. 2023 Sep 17;33(50):2301857. doi: 10.1002/adfm.202301857 (PMC10941700; doi:10.1002/adfm.202301857)
Supplement: Supplementary file 1 — Supporting Information [file ADFM-33-2301857-s001.pdf]

ADVANCED FUNCTIONAL MATERIALS

Supporting Information

for *Adv. Funct. Mater.*, DOI 10.1002/adfm.202301857

Low-Field Actuating Magnetic Elastomer Membranes Characterized using Fibre-Optic Interferometry

Zhi Li, Joanna. M. Coote, Swathika Subburaman, Francesco Iacoviello, Kristopher Page, Erwin J. Alles, Polina Prokopovich, Ivan P. Parkin, Adrien E. Desjardins and Sacha Noimark*

Supporting Information

Low-field actuating magnetic elastomer membranes characterized using fiber-optic interferometry

Zhi Li, Joanna. M. Coote, Swathika Subburaman, Francesco Iacoviello, Kristopher Page, Erwin J. Alles, Polina Prokopovich, Ivan P. Parkin, Adrien E. Desjardins, Sacha Noimark

This supporting information includes:

Figure S1. Photographs of cured ME (magnetic elastomer) membranes and assembly probe
Figure S2. Surface roughness analysis of ME membranes
Figure S3. ANSYS modelling of magnetic displacement of the ME membrane
Table S1. Theoretical calculation of magnetic deflection of the ME membrane
Figure S4. Background noise of the fibre-optic interferometric system
Figure S5. Magnetic response speed study of ME membranes
Figure S6. Fast response measurement of ME membranes
Figure S7. Field modulated magnetic response of ME membranes
Figure S8. Contact angle (water) measurements of ME membranes
Figure S9. Magnetic field distribution of the voltage modulated electromagnet

Figure S1. Photographs of a) cured ME (magnetic elastomer) membranes of various concentrations (backing with yellow Kapton tape), b) cured ME 60% within single-layer Kapton template, c) glass ferrule (cone diameter of 880 μm) attachment on cured ME 60%, d) fabricated ME 60% on different structures: small square acrylic frame, circular glass petri dish, and large circular acrylic frame (inner diameter of 200 mm), e) ME membrane-ferrule assembly was integrated with SM fibre ended with a FC/APC connector, f) fibre-membrane-ferrule assembly under high magnification.

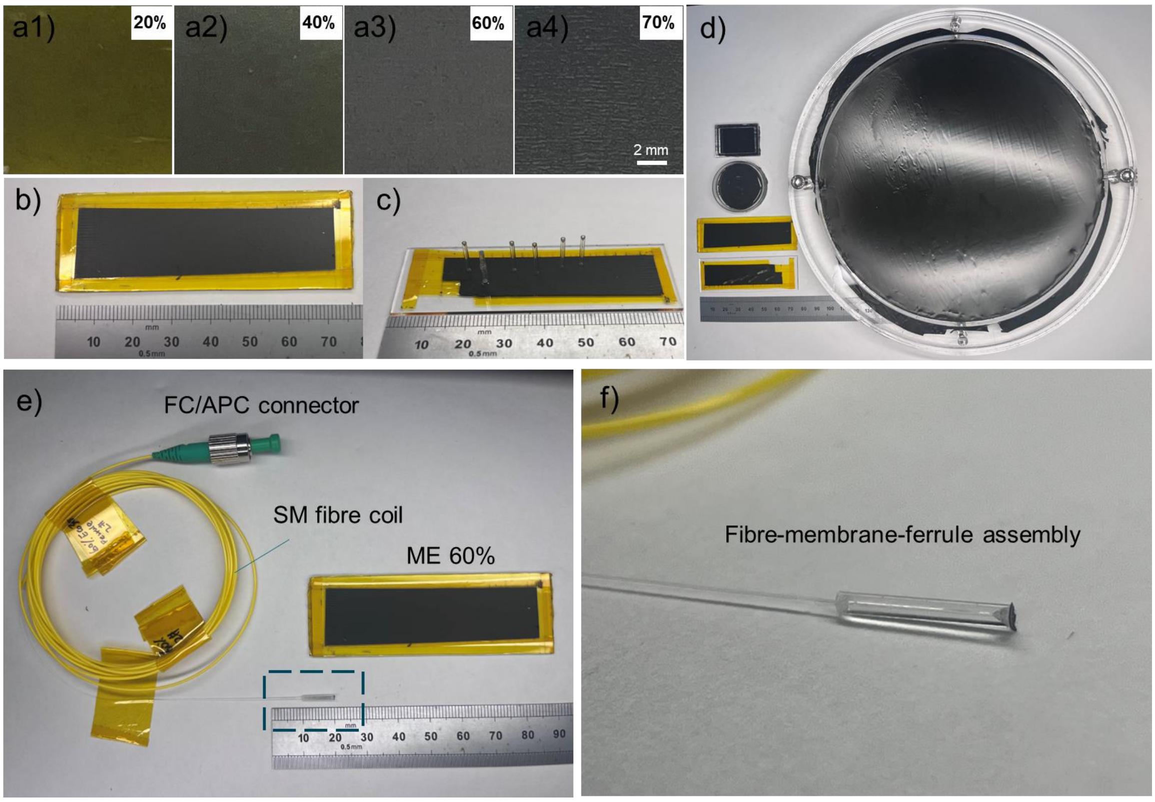

Figure S2. Surface roughness analysis of ME membranes using DektakXT® StylusProfiler (Bruker, USA). a-d) Camera screenshots of surface profile scans (Vision64™ system). e) Roughness profile, f) average surface roughness of ME membranes. Surface scans were performed over a scan length of 500 µm with a stylus tip of diameter 5 µm, a scan range of 6.5 µm, and a scan resolution of 0.0333 µm/point. Slow speed (500 µm in 50 s) and low stylus force of 0.3 mg were employed to avoid tip bouncing or sliding on a soft substrate and to ensure no scratches or damages over the membrane surface during contact mode scanning.

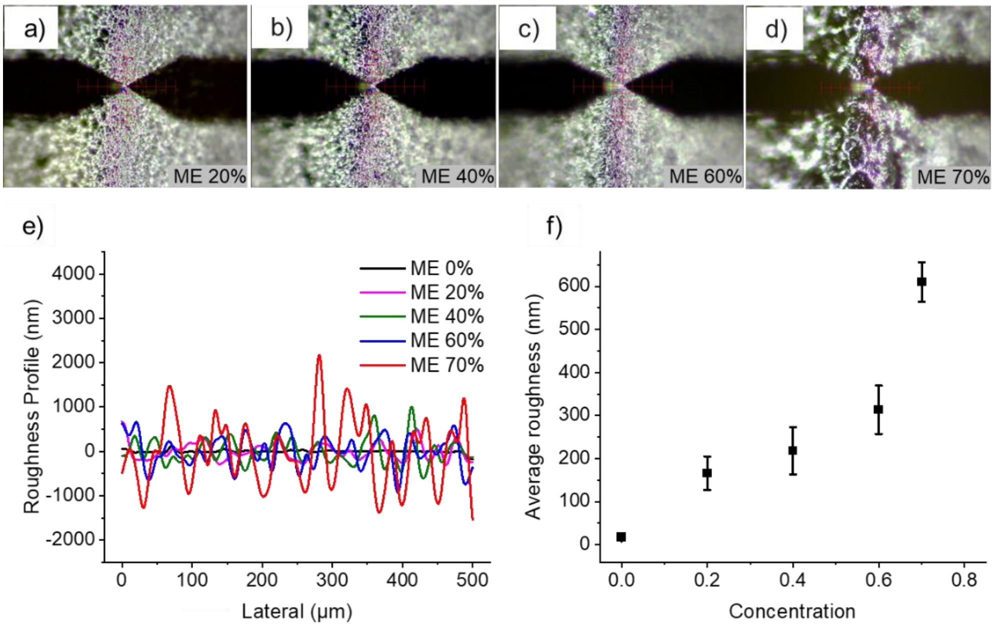

Screenshots of the scan camera (Figure S2a-d) showed that the surface morphology of the ME membranes at a magnification remained unchanged when particle loading was less than or equal to 60 %. However, ME 70% showed a markedly different morphology, with large clusters of particles clearly observed. The profile scans in Figure S2e showed that an increased particle loading corresponded to an approximately linear increase in surface roughness up until ME 70%, at which point the elastomer surface integrity deteriorated with a significant increase in roughness. The average surface roughness was calculated to be 17.2 nm, 166.2 nm, 218.2 nm, 313.6 nm, and 610.1 nm for pure Ecoflex, ME 20%, ME 40%, ME 60%, and ME 70%. A significant increase in surface roughness was statistically confirmed as the magnetic particle loading increased up to 70%.

In this work, the membrane displacement was measured with the interferometric sensing system that involved optical reflection from the membrane surface. Theoretically, a smooth surface with low roughness promotes stronger reflection and spatial coherence, resulting in a higher signal-to-noise ratio and greater sensitivity. However, in this work, we did not observe any significant differences in optical reflectivity when ME membranes of different loadings (0%-70%) were tested. Future work will focus on improving optical specular reflection, e.g., by creating a sandwiched structure with highly reflective materials (e.g., Au) on the ME membrane surface.

Figure S3. ANSYS simulation result showing a) the top view and b) the horizontal view of a circular and edge-supported ME membrane (ME 60%-L1-D880) actuated under a magnetic field (driving voltage: 3V) with a field amplitude of 8.3 mT and field gradient of 740 mT/m.

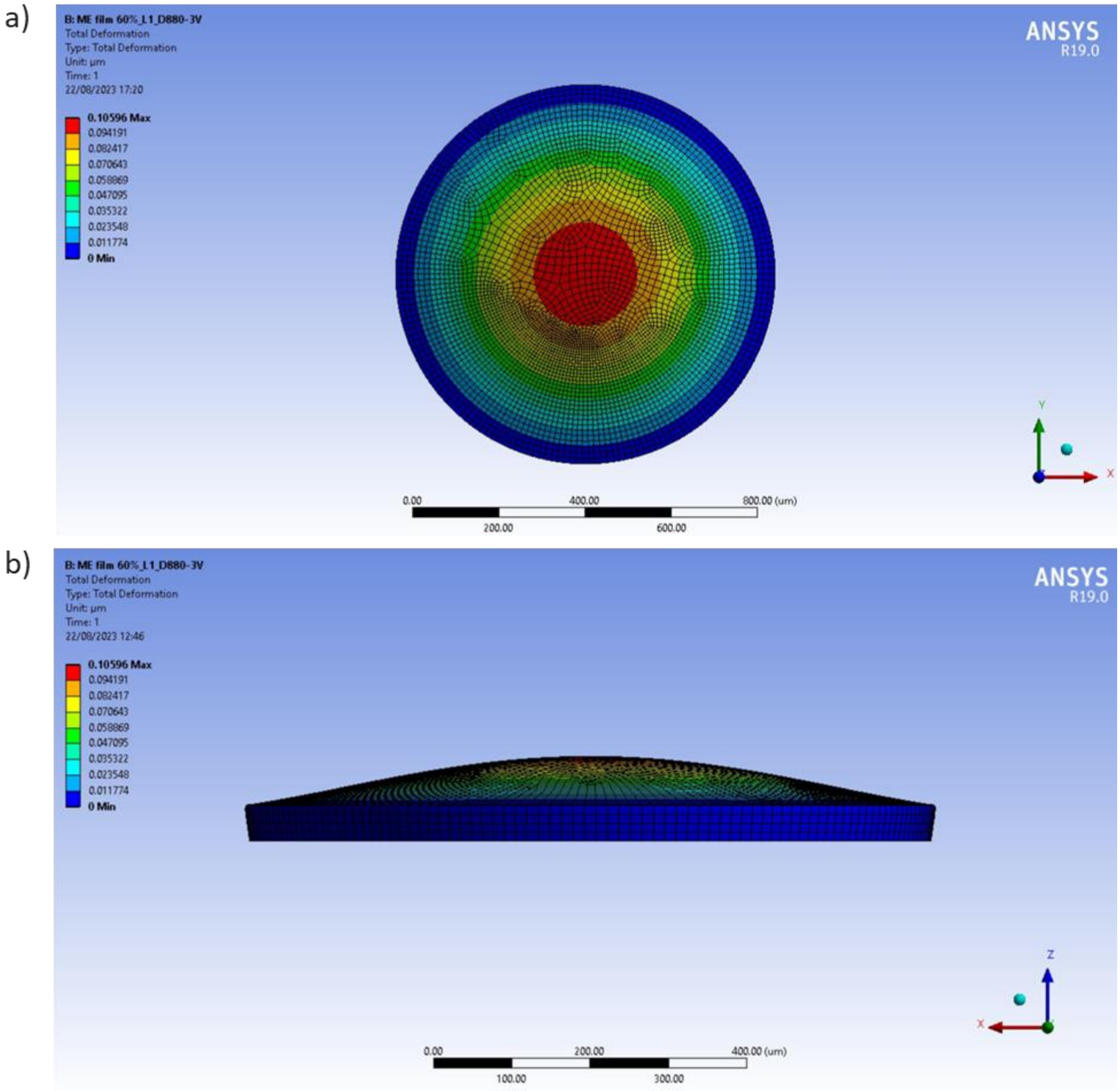

The ANSYS modelling parameters were listed in the table below. Material model was set to mimic the membrane conditions. A fixed edge was chosen as the boundary support to allow deflection of the free-standing membrane. The mesh size was chosen as 15 µm adaptively across the whole body. The perpendicular component of the magnetic force was theoretically calculated and uniformly applied to the surface of the ME membrane; it formed a magnetic pulling pressure loading (p_z) given by:

$$F_z = \mathrm{V}\mu_0 \frac{\Delta H_z}{\Delta z} M_{membrane}$$

$$\mathrm{p_z} = \frac{F_z}{s} = \frac{t \cdot F_z}{V}$$

where V is the effective volume of the membrane, μ_0 is the vacuum magnetic permeability ($4{\cdot}10^{7}$ T·m·A^{-1}), $\frac{\Delta H_z}{\Delta z}$ is the field gradient (740 mT/m), and $M_{membrane}$ is the volume magnetization given by:

$$M_{membrane} = H \cdot \chi_V$$

H is the external magnetic field (8.3 mT), and χ_V is the volume susceptibility (1.0744 $emu/(cm^3 \cdot$ mT). The magnetic force (F_z) was calculated to be 0.181 µN, and the loading pressure (p_z) was then obtained as 0.297 Pa.

According to the simulation results, the magnetic deflection was symmetrically distributed along the radial direction across the membrane. It attained a maximum of 106 nm at the centre of the membrane and decreased rapidly with increasing distance from the centre point until it reached zero at the fixed edge. ANSYS simulations provided an approximate graphic estimation of the magnetic displacement of a circular and free-standing ME membrane with a specific concentration and size.

Table 1. Modelling parameters of ANSYS simulation

**Modelling setting**		**Description**	**Quantity**	**Unit**
**Material data**	ϕ	Particle concentration	60	%
	E	Young's modulus	320	kPa
	ν_0	Poisson's ratio	0.49	-
	ρ	Density	1.983	g/cm^3
**Model geometry**	d	Diameter	880	µm
	t	Thickness	45	µm
**Boundary condition**		Fixed edge support		
**Loading condition**	B	Magnetization	8.3	mT
	$\frac{\Delta Hz}{\Delta z}$	Gradient along z direction	740	mT/m
	F	Magnetic pulling force	0.181	µN
	p	Pressure per surface area	0.297	Pa
**Mesh**	a	Mesh size	15	µm

Table S1. Theoretical calculation of the magnetic deflection of a circular and edge-clamped membrane (ME 60%-L1-D880) under a 3V magnetic field (8.3 mT, 740 mT/m).

Parameters	Description	Quantity	Unit
ϕ	Particle concentration	60	%
d	Diameter	880	μm
E	Young's modulus	320	kPa
t	Thickness	45	μm
B	Magnetization	8.3	mT
$\frac{\Delta \mathrm{Hz}}{\Delta \mathrm{z}}$	Gradient along z direction	740	mT/m
F	Magnetic pulling force	0.181	μN
ω_1	Theoretical deflection (Edge simply supported)	200	nm
ω_2	Theoretical deflection (Edge clamped)	54	nm

The maximized displacement of an edge simply supported membrane can be calculated by following[1]:

$$\omega_0 = \frac{pr^4(5+\nu_0)}{64G(1+\nu_0)}$$

in which p is the magnetic force pressure (N/m^2), r is the effective radius of the deflecting membrane (r= d/2, d is the diameter of the membrane), G is the flexural rigidity, ν_0 is the Poisson's ratio assumed to be a constant value 0.49 for all studied ME membranes.

Discrepancies between the membrane displacements obtained from theoretical calculations and the ANSYS simulations and those observed experimentally may have arisen from differences in the assumed mechanical boundary conditions. In theoretical calculations and ANSYS simulations, the membrane was either edge clamped or simply supported; however, these two conditions likely do not fully capture the mechanical properties of the ME membrane.

Figure S4. Background noise of fibre-optic interferometric sensing system during a real-time displacement measurement of ME 60% (thickness of 43 µm and diameter of 880 µm) in the absence of magnetic field. Standard deviation of the noise was calculated as 2.495 nm, which determines the limit of detection of the sensing system.

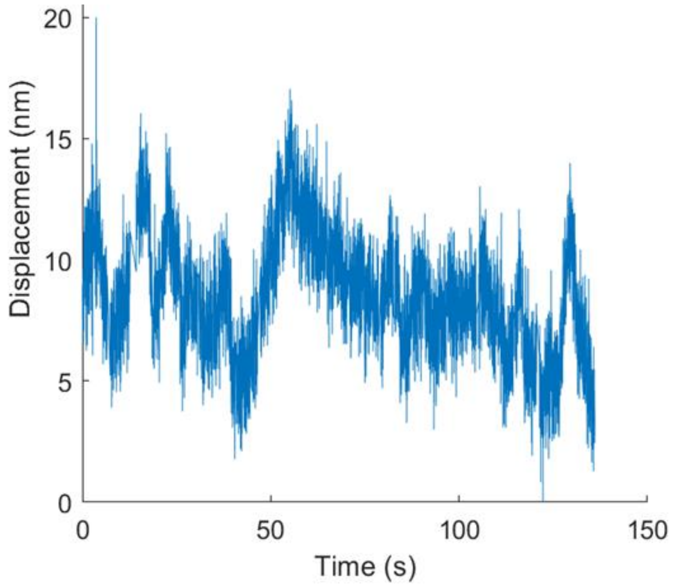

Figure S5. Magnetic response time analysis of ME 60% (thickness of 43 µm and diameter of 880 µm) under a 3V magnetic field. Data from the initial response stage was acquired and analysed.

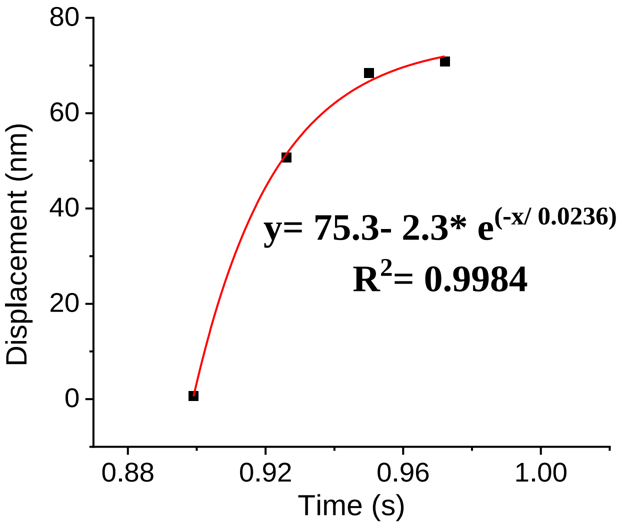

Exponential fitting curve ($y = a + b*\exp(-t/\tau)$) of the initial response stage was plotted to obtain a time constant (τ= 0.0236 s), which can be regarded as the response time of the ME membrane.

Figure S6. Fast response measurement of ME 60% (thickness of 43 μm and diameter of 2000 μm). A range of pulse cycle periods was employed as the applied on-off field with an amplitude of 1V (3.3 mT, 280 mT/m).

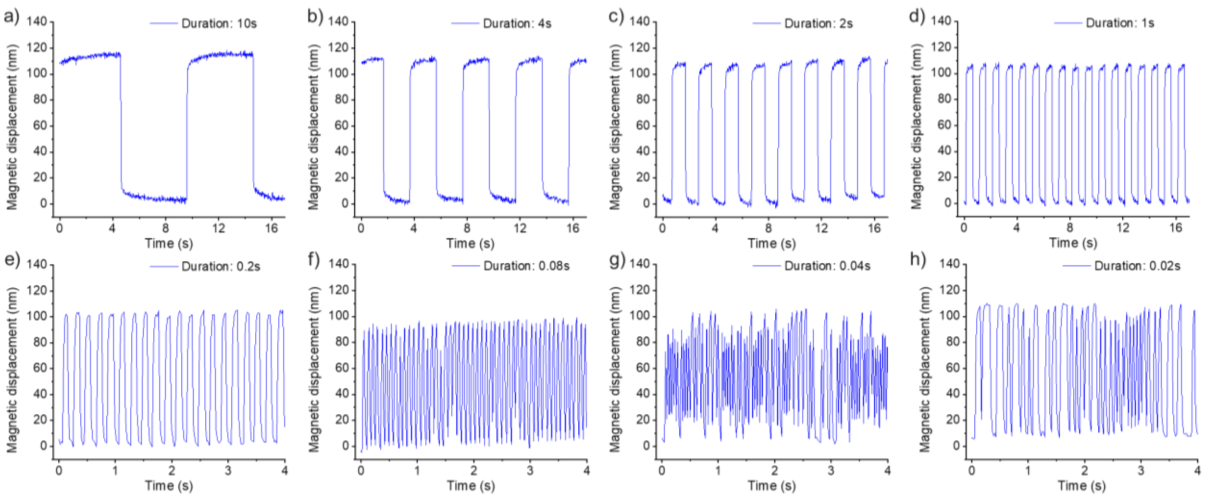

As seen in the figure above, magnetic displacement was consistently recorded and maintained (around 108 nm) in a fast manner as the pulse cycle period was reduced from 10 s down to 0.2 s (5 Hz). Higher excitation frequency (0.08 s, 12.5 Hz) caused a slight reduction of magnetic displacement as it had not reached the fully stabilized level (the second quasi-equilibrium stage) before recovering. Further increase in excitation frequency caused discontinuous and distorted displacement recordings due to the failure of fast field generation and limited data acquisition rate.

Figure S7. Field modulated magnetic response of ME 60% (thickness of 43 µm and diameter of 2000 µm). The black and red lines represent the magnetic displacement curves either under the regular square-wave pulse field (both pulse interval and duration are 1s) or under a stepwise varying field (100 stepwise change within 1s for each ascending or descending phase). Note that those two modulated responses (black and red) were displayed together in each figure but not synchronized in time. 1V, 3V, 4V, 5V, 6V and 8V correspond to the field strength of 3.3 mT, 8.5 mT, 10.8 mT, 13.3 mT, 15.8 mT and 21.5 mT, respectively.

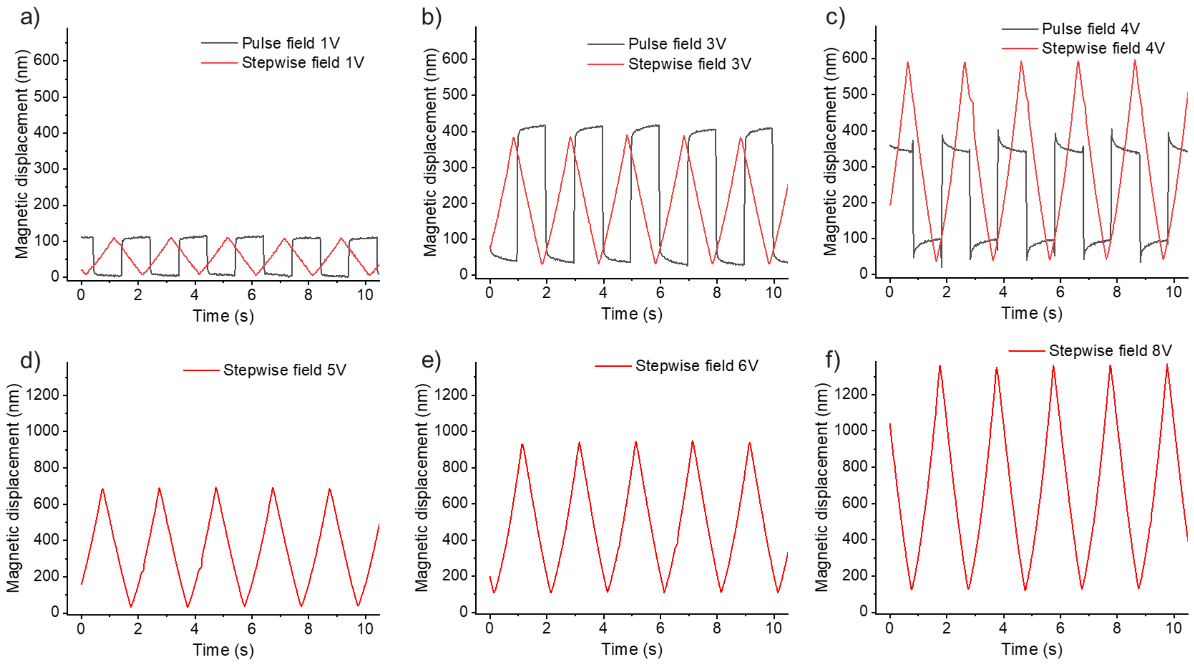

As seen and indicated by the black lines in a-c), the phase wrapping effects appeared under the modulation of the on-off pulse field over 4V (10.8 mT, 940 mT/m). Therefore, discontinuous recording may happen under the on-off field modulation when field induced displacement exceeds the limit. However, under the modulation of stepwise varying field (red line) as shown in a-f), the magnetic actuation and optical monitoring of the ME membrane were successfully demonstrated in a wide range of magnetic fields (up to 8V) and displacement levels (more than 1000 nm). Despite distinct modulation fields, the induced displacements were measured to be surprisingly close, which exhibits good consistency and reproducibility of the measurements.

Figure S8. Contact angle measurement (water) of ME membranes (0, 10, 40, 60 and 70 wt.\%). Measurements were characterized using a Krüss DSA 25E drop shape analyser (KRÜSS GmbH, Hamburg, Germany). Water drops were dispensed onto the test substrates using a liquid needle double-dosing unit, and angles were computed in Krüss Advance software. Raw magnetite powder and the ME membranes, including pure Ecoflex (0 wt.\%), showed similar contact angles around 107° (> 90°) regardless of particle loadings, which indicated hydrophobic properties.

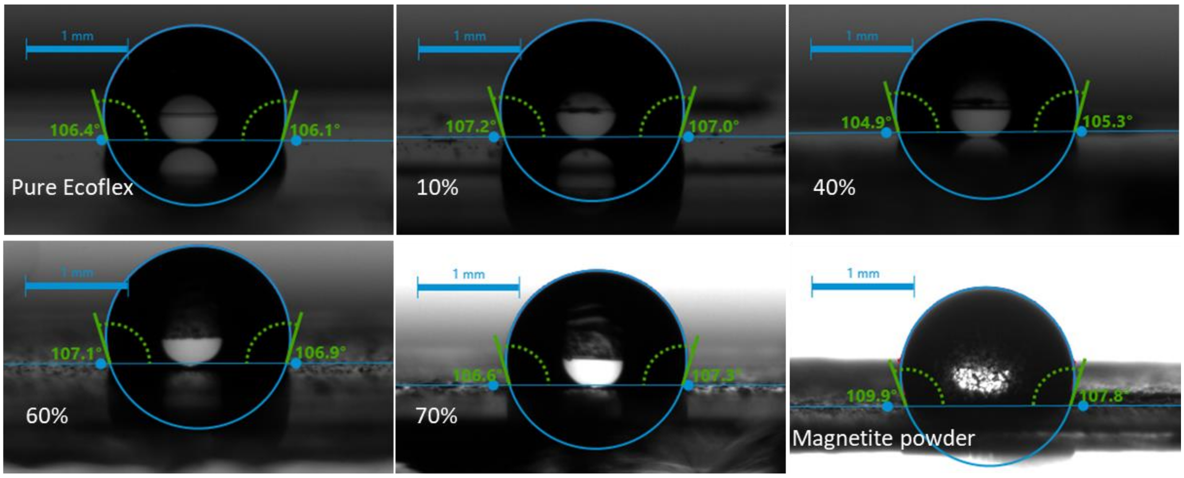

Figure S9. Magnetic field mapping of the electromagnet under different voltages. a) Schematic illustration of the field measurement. The gaussmeter probe was placed centrally over the electromagnet and translated along the z-axis (the sampling distance interval was set to be 500 μm). b) Magnetic gradient and c) field strength distribution under various applied voltages.

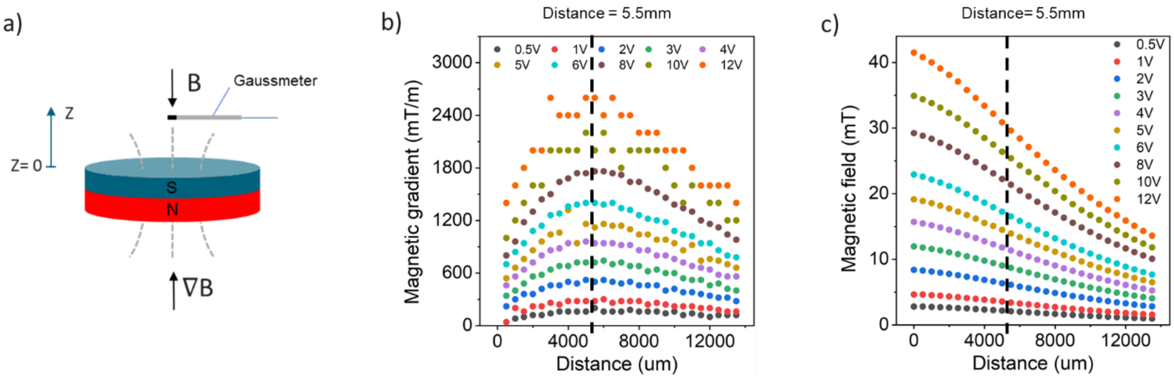

As shown in b) and c), the field gradient maximized with minor variations at around 5.5 mm away from the electromagnet, where the field strength showed good linearity. Therefore, in this work, the membrane-ferrule assembly probe was fixed and placed 5.5 mm away from the magnet in all tests to minimize the errors of the field induced by spatial variations.

Reference

[1] S. Timoshenko, S. Woinowsky-Krieger, B. Guatemala, H. Lisbon, L. Madrid, M. New, D. Panama, P. San, J. Sso, P. Singapore, S. Tokyo, *Theory of Plates and Shells*, 2nd ed., Mcgraw-Hill Book Company, **1959**.
